# Charles bonnet syndrome with subsequent depression in acute angle-closure glaucoma: a case report

**DOI:** 10.1093/omcr/omag024

**Published:** 2026-03-23

**Authors:** Priyanka Renita D’Souza, Adarsh Sugathan

**Affiliations:** Department of Psychiatry, Kasturba Medical College Mangalore, Manipal Academy of Higher Education, Manipal, India; Department of Internal Medicine, Manipal Hospitals, KMC Hospital, Jyothi Circle, Mangalore, Karnataka 575001, India

**Keywords:** Charles bonnet syndrome, glaucoma, visual hallucinations, depression

## Abstract

Charles Bonnet syndrome (CBS) presents with complex visual hallucinations in visually impaired individuals with preserved insight. While most commonly associated with macular degeneration, it may occur in glaucoma. CBS is often under-reported due to stigma and may progress to psychiatric morbidity. A 63-year-old man with bilateral visual loss following acute angle-closure glaucoma experienced vivid hallucinations of animals and snakes. Initially concealed due to embarrassment, the symptoms were revealed during psychiatric assessment. Ophthalmic evaluation confirmed glaucomatous changes; MRI brain was recommended but declined. CBS was diagnosed, and hallucinations resolved with reassurance and low-dose clonazepam. Several weeks later, the patient developed moderate depression (HAM-D = 18), successfully treated with mirtazapine. At three months, he remained asymptomatic. This case is unusual for its association of CBS with angle-closure glaucoma and subsequent depression, underscoring the need for proactive inquiry, de-stigmatization, and integrated ophthalmic–psychiatric care.

## Introduction

Charles Bonnet syndrome (CBS) is a disorder that causes intense, sophisticated visual hallucinations in people with visual impairment but with intact cognitive functions and insight about the unreal aspect of visual hallucinations. Despite its prevalence among the elderly and those with degenerative eye disorders, CBS goes undiagnosed and untreated due to a lack of understanding and misconceptions about its etiology and management [1].

The 18th-century Swiss naturalist Charles Bonnet is credited with the earliest description of the phenomena known as Charles Bonnet syndrome. Although the etiology of CBS is obscure, two of the most popular explanations for the origins of CBS hallucinations have garnered a lot of attention in the literature. The "release theory" suggests that visual pathway lesions cause abnormal signals to the visual cortex [2] and the "deprivation theory" posits that decreased sensory input, such as sub-threshold visual inputs, causes the visual association cortex to form spontaneous images, leading to visual hallucinations [3]. Despite being a common condition among the elderly and people with visual defects, the challenge is in the diagnosis and management. Due to limited awareness, CBS gets ignored and less frequently diagnosed. In addition, the stigma towards psychiatric illness poses a diagnostic challenge. It is of due importance to understand the psychological impact of visual deficits and CBS as it has a negative impact on the quality of life of an individual. Therefore, our case report of CBS and depression in an individual with glaucoma highlights the need for a holistic approach with a multidisciplinary team in glaucoma.

## Case report

A 63-year-old man presented with reduced vision and vivid visual hallucinations of brightly coloured animals and snakes lasting several minutes, occurring multiple times daily and abating with eye closure. He retained insight and denied auditory hallucinations or other psychotic features. Initially he concealed symptoms from family members and clinicians because of embarrassment.

Fifteen days earlier he had undergone bilateral Neodymium-doped Yttrium Aluminium Garnet (Nd:YAG) peripheral iridotomy for acute angle-closure glaucoma. Visual acuity was 6/24 (right eye) and 6/18 (left eye). Examination revealed corneal oedema, shallow anterior chambers, immature cataracts, a cup–disc ratio of 0.5, and a left macular dot-blot haemorrhage. Intraocular pressures were unrecordable at presentation; anterior segment optical coherence tomography confirmed narrow/closed angles. Magnetic resonance imaging (MRI) of the brain was advised to exclude structural lesions but was declined by the patient.

On psychiatric evaluation, orientation and cognition were intact. Given the preserved insight, the exclusively visual nature of the hallucinations, and the temporal relationship to acute visual loss, a clinical diagnosis of Charles Bonnet Syndrome was made. The patient received reassurance and psychoeducation, and was started on low-dose clonazepam (0.25 mg at night) for anxiety associated with hallucinations. The visual hallucinations resolved over the subsequent four weeks.

At follow-up he developed persistent low mood, insomnia, and hopelessness. Hamilton Depression Rating Scale (HAM-D) score was 18 (moderate depression). He was initiated on mirtazapine 15 mg daily and received brief supportive counselling along with referral for low-vision rehabilitation. Depressive symptoms improved over six weeks; at three months there was complete resolution of hallucinations and depressive symptoms.

## Discussion

CBS is very often under-diagnosed despite its high prevalence, especially in the elderly. The risk factors of CBS are also not clearly defined; however, possible associations have been reported. In our case report, glaucoma was associated with CBS which is in conjunction with a case study reported in Sweden [4]. However, the psychological impact of visual field loss was not discussed. Head trauma has also been noted to trigger CBS in another case study reported in Washington [5]. One more case with an elderly female diagnosed with right eye indirect traumatic optic neuropathy had features of CBS with images of children [6]. The patient’s refusal for MRI brain became a limitation for the case. Nevertheless, alternative causes were considered and clinically excluded: psychotic depression was unlikely given the absence of pervasive low mood along with visual hallucination in the initial presentation; peduncular hallucinosis, which classically follows brainstem/thalamic lesions and is usually accompanied by other brainstem signs or prominent sleep–wake disturbance and other structural lesions, was not supported by examination or history; and dementia-related visual hallucinations were considered less likely because cognition was preserved on psychiatric assessment, functional status remained intact, and symptoms resolved quickly with conservative measures. The temporal correlation with acute visual loss and preserved insight supported a clinical diagnosis of Charles Bonnet Syndrome despite the absence of neuroimaging.

With various definitions of CBS stated in current literature, it is uncertain if the diagnostic criterion for CBS includes a normal neuropsychiatric assessment and the absence of dementia. Subclinical Alzheimer's or Lewy Body pathology may play a role in CBS; however, this remains unclear. Case studies have linked CBS to a later diagnosis of Alzheimer's or Lewy body dementia [7]. CBS lacks well-established diagnostic criteria and standard screening tools. But, International Classification of Diseases (ICD) 11 has a new development in this area with visual release hallucinations included under the category 9D56 [8].

The symptoms of CBS are a complex of ophthalmic and neuropsychiatric and therefore there is no standard line of management established. A comparison of similar reported cases is shown in Table 1 [9–11]. Unlike most reports, our patient developed moderate depression following CBS, an uncommon trajectory that required pharmacological intervention with Mirtazapine on follow up. Due to its 5-HT2A neurotransmission-blocking properties and its effective administration in prior studies with CBS, Mirtazapine was the preferred drug in this case, despite initial trial of Clonazepam [11]. The postulated mechanisms of depression as a sequelae to CBS are multifactorial. They may include sensory deprivation, which leads to reduced input to the visual cortex and subsequent release hallucinations; cortical disinhibition with spontaneous hyperactivity in visual association regions and neurobiological overlap of decreased serotonin levels in sensory-deprived cortex; and significant psychological distress triggered by the adjustment reaction to potential blindness [12]. Cox and Ffytche conducted a study on the negative outcome of CBS and noted that one-third of medical professionals were either uncertain or unaware of CBS and its symptoms [13]. A review by Jones et al found that lack of awareness of CBS may worsen patients' anxiety. The stigma associated with visual hallucinations which was also observed in this case may have a negative impact on CBS patients' ability to seek help and share symptoms, as well as worsen isolation and feelings of loneliness [14, 15]. Therefore, our first line of management included reassurance and psychoeducation of the patient in this case.

**Table 1:** Few selected cases of Charles bonnet syndrome with psychiatric manifestations.

Author & Year	Underlying eye disease	Hallucination features	Psychiatric sequelae	Management	Outcome
Teunisse et al., 1996 [9]	Macular degeneration	People, faces	None	Reassurance	Spontaneous remission
Pang, 2016 [10]	Diabetic retinopathy	People, landscapes	Depression	Supportive	Improved with vision stabilization
Risi et al, 2023 [11]	Glaucoma	Snakes, Bugs	Delusional Parasitosis, Anxiety	Supportive counselling, anxiolytics, Mirtazapine	Improved
Present case, 2025	Acute angle-closure glaucoma	Multicolored animals, snakes	Moderate depression (HAM-D 18)	Reassurance (initial), Clonazepam + mirtazapine	Hallucinations resolved, depression remitted

Since most of the focus is on visual rehabilitation, the psychological implications are not well explored in glaucoma. Due to the irreversible nature of blindness, patients may have difficulty coming to terms with the diagnosis of glaucoma. Therefore, the management should comprehensively involve treating the visual field defects as well as screening for psychological disorders like depression and anxiety.

Nonetheless, awareness of CBS is limited, particularly among healthcare professionals and low-vision patients. The barrier of delayed disclosure due to stigma, and progression to depression in this case emphasize that CBS is not always benign and requires multidisciplinary care. The utilization of standardized tools for screening of psychiatric manifestations like depression or anxiety may help in the holistic management. A tiered management strategy is indicated, with education relating to CBS and reassurance in individuals with visual deficits. Therefore, this case report emphasizes the importance of physician and patient education and training in facilitating a proactive approach to CBS and glaucoma management.
